# Personal

**Published:** 1905-03

**Authors:** 


### PERSONAL.

DR. CHARLES A. L. REED, of Cincinnati, has been appointed a member of the boundary commission to determine the limits of the Isthmian canal zone. He sailed for Panama, January 31, 1905.

---

DR. E. C. W. O'BRIEN, surgeon of the Buffalo fire department, who has been seriously ill with grip and its sequelæ, was reported improved, but subsequently suffered a relapse. Dr. O'Brien's many friends who sympathise with him in his prolonged illness are hopeful of his ultimate recovery.

---

DR. WILLIAM G. RING, of Buffalo, has been appointed surgeon of the fire department, ad interim, pending the recovery of Dr. O'Brien.

---

DR. MARTIN BESSEMER, of Ithaca, has been appointed by the regents of the University of the State of New York a member of the homeopathic state board of medical examiners, vice Dr. William L. Fiske, of Brooklyn, deceased.

---

DR. WILLY MERCK, member of the house of E. Merck, Darmstadt, has received from the University of Halle, Germany, the honorary degree of doctor of medicine, conferred in recognition of numerous contributions to therapeutics.

---

DR. DEWITT G. WILCOX, of Buffalo, was elected president of the Homeopathic Medical Society of the State of New York at its annual meeting held at Albany, February 14 and 15, 1905.

